# Quality of Life Assessment in Female Rheumatoid Arthritis Patients

**DOI:** 10.1192/j.eurpsy.2024.1687

**Published:** 2024-08-27

**Authors:** A. Feki, I. Sellami, I. Mnif, Z. Gassara, S. Ben Djemaa, A. Abbes, M. Ezzeddine, M. H. Kallel, H. Fourati, R. Akrout, S. Baklouti

**Affiliations:** ^1^Rheumatology, Hedi Chaker Hospital; ^2^ Medicine university; ^3^Occupational medicine, Hedi Chaker Hospital, Sfax, Tunisia

## Abstract

**Introduction:**

Rheumatoid arthritis (RA) is a chronic inflammatory disease that significantly impacts patients’ quality of life (QOL), affecting both physical and mental well-being. QOL is predictive of morbidity and mortality, making its consideration increasingly important in treatment decisions.

**Objectives:**

This study aims to assess the Quality of Life in Female Rheumatoid Arthritis patients.

**Methods:**

The study included 87 female patients with confirmed RA, diagnosed by an experienced rheumatologist based on the ACR 1987 or ACR/EULAR 2010 criteria. Quality of life was assessed using the World Health Organization Quality of Life assessment, short form (WHOQOL-BREF scale). The scoring ranged from 0 to 100 for each domain. Disease activity was assessed using the Disease Activity Score (DAS28), and functional disability was evaluated using the Health Assessment Questionnaire (HAQ).

**Results:**

The study included 87 patients with a mean age of 54.7 ± 12.2 years and a mean disease duration of 12 ± 9.1 years. The majority of patients had a medium socioeconomic level (81.6%), and a low cultural level with 31% being illiterate, 6% attending university, and 76.9% unemployed. Regarding marital status, 74.7% were married. RA was erosive in 77% of patients, deforming in 68%, and 40% were seropositive (FR and/or anti-CCP). Extra-articular manifestations were present in 34.5% of patients. Sixty-seven patients (77%) were on disease-modifying antirheumatic drugs (DMARDs), with 67.8% on methotrexate. Eighteen percent were treated with biological agents. Corticosteroids were used by 47.1% of patients, while 12.6% used non-steroidal anti-inflammatory drugs, and 6.9% used both. Disease activity varied, with 9.2% having low activity, 43.7% moderate activity, and 24.1% high activity based on DAS28. The mean HAQ index was 1.1 ± 0.8, indicating moderate to severe disability for more than 60% of patients. The mean WHOQOL scores were substantially reduced in the physical health (43 ± 16.2), psychological health (50.3 ± 14.4), social relationships (51.5 ± 18.6), and environment domains (46.8 ± 15). There was a significant inverse correlation between HAQ and the physical health (r = −0.52, p < 0.001), psychological (r = −0.57, p < 0.001), social relationships (r = −0.37, p = 0.001), and environmental domains (r = −0.45, p < 0.001) of QOL. There was no correlation between any domain of QOL and DAS28.

**Conclusions:**

Patients with RA experience reduced QOL across multiple domains, including physical function, mental health, and social relationships. Functional disability, as reflected by HAQ, is the most significant factor affecting QOL in RA. The WHOQOL-BREF should be considered a valid outcome measure for interventions aimed at improving the quality of life for people with rheumatoid arthritis.

**Disclosure of Interest:**

None Declared

